# Assessment of Psychosocial Programs to Prevent Sexual Violence During Adolescence: A Systematic Review and Meta-analysis

**DOI:** 10.1001/jamanetworkopen.2022.40895

**Published:** 2022-11-08

**Authors:** Antonio Piolanti, Ernest N. Jouriles, Heather M. Foran

**Affiliations:** 1 Health Psychology Unit, Institute of Psychology, Universität Klagenfurt, Klagenfurt, Austria; 2 Department of Psychology, Southern Methodist University, Dallas, Texas

## Abstract

**Importance:**

Sexual violence is a public health problem that affects adolescents globally. To our knowledge, no meta-analysis of prevention programs for adolescent sexual violence has been conducted.

**Objective:**

To perform a systematic review and meta-analysis of randomized clinical trials evaluating the efficacy of psychosocial programs for preventing sexual violence during adolescence.

**Data Sources:**

Peer-reviewed articles published in English were searched in PsycINFO, ERIC, PsycArticles, PubMed, and Web of Science databases through December 2021.

**Study Selection:**

Studies were included if they were randomized clinical trials assessing the efficacy of a psychosocial prevention program targeting sexual violence and delivered to adolescents aged 10 to 19 years.

**Data Extraction and Synthesis:**

Preferred Reporting Items for Systematic Reviews and Meta-analysis (PRISMA) guidelines were followed to identify studies. The quality of individual studies was assessed with the Revised Cochrane Collaboration Risk of Bias tool. A random-effects model was used to pool odds ratios (ORs). Exploratory subgroup and metaregression analyses were performed to evaluate the associations between moderators and effect sizes.

**Main Outcomes and Measures:**

Primary outcomes were perpetration of sexual violence, experience of sexual violence, and a composite measure of any perpetration or experience of sexual violence.

**Results:**

Data were analyzed from 20 trials involving 37 294 adolescents. Compared with control conditions, prevention programs were associated with a significant reduction in the perpetration (OR, 0.83; 95% CI, 0.73-0.95; *P* = .005) and experience (OR, 0.87; 95% CI, 0.78-0.98; *P* = .02) of sexual violence, as well as a 13% significant reduction of any sexual violence (OR, 0.87; 95% CI, 0.78-0.97; *P* = .009). Exploratory analyses of the combination of program setting and participant age indicated that programs that were delivered in school settings and targeted at adolescents aged 15 to 19 years yielded significantly larger effect sizes (Cochran Q = 4.8; *P* = .03) compared with programs that were either delivered outside of a school setting or targeted younger adolescents. Quality assessment of trials revealed concerns of risk of bias across several included studies.

**Conclusions and Relevance:**

In this meta-analysis, evidence suggested that prevention programs were associated with reducing adolescent sexual violence, especially when implemented at school with older adolescents. However, there is need for additional high-quality research. Prevention of adolescent sexual violence remains understudied compared with other similarly important public health prevention targets.

## Key Points

**Question:**

Are psychosocial prevention programs associated with reduced sexual violence during adolescence?

**Findings:**

In this meta-analysis of 20 randomized clinical trials that included data from 37 294 adolescents, prevention programs were associated with a significant reduction in perpetration and experience of sexual violence, as well as a composite measure of any sexual violence, during adolescence.

**Meaning:**

These findings suggest the potential efficacy of prevention programs for reducing adolescent sexual violence.

## Teaser

This meta-analysis of 20 trials examines the use of prevention programs to reduce sexual violence during adolescence.

## Introduction

Sexual violence is often defined to include a wide range of sexual acts—from forced kissing to rape— in which consent is not obtained or freely given.^1^ Across the world, adolescents aged 10 to 19 years old have experienced (ie, been the target of) such violence.^2,3^ National surveys in the US indicate that between 10% to 20% of US adolescents have reported experiencing past-year sexual violence,^4^ with comparable rates found in other countries.^5^ Some of the sexual violence directed at adolescents is perpetrated by adults, but much of it is committed by other youth. For example, in a national sample of US youth, 10% of adolescents reported they had perpetrated some form of sexual violence during their life.^6^ Youth who are the target of sexual violence are at risk for many short- and long-term harmful outcomes, including mortality, morbidity, and a wide range of psychiatric symptoms.^7,8,9^ Youth who perpetrate sexual violence also report problematic outcomes.^10^ The prevalence and harmful outcomes associated with sexual violence during adolescence make its prevention a high priority.

Prior reviews of prevention programs for youth have focused on sexual violence in the context of intimate partner violence,^11,12^ thus excluding programs designed to prevent sexual violence outside of adolescent romantic relationships. The reviews suggest that, overall, the prevention programs evaluated thus far may not be effective in reducing sexual violence. For example, a meta-analysis on dating violence reported that prevention programs that seemed to reduce physical violence did not show effects on sexual violence.^12^ Similarly, a systematic review on prevention programs for sexual violence perpetration in adults and youth did not find clear evidence supporting the efficacy of such programs.^13^ The latter review did not consider youth separately from adults, and it focused only on perpetration.

The past decade has witnessed an upsurge of psychosocial programs to prevent youth from perpetrating sexual violence and being the target of such violence. Notably, these programs are delivered with a variety of formats. For example, some programs are delivered in the context of educational activities during school, whereas others are offered outside of school settings; some target both boys and girls, whereas others target a single gender. To date, there has been no systematic attempt to empirically evaluate the overall efficacy of psychosocial programs for preventing sexual violence during adolescence or to determine if effectiveness varies as a function of programs’ characteristics. The primary aim of the current study was to conduct a systematic review and meta-analysis of randomized clinical trials (RCTs) that have evaluated programs designed to reduce adolescent sexual violence. The review also addressed questions about the differential effectiveness of programs with different delivery formats.

## Methods

### Search Strategy

The meta-analysis and systematic review was conducted according to the Preferred Reporting Items for Systematic Reviews and Meta-analyses (PRISMA) reporting guideline.^14^ First, PsycINFO, ERIC, PsycArticles, PubMed and Web of Science databases were searched from inception through August 2021 using English search terms. We used a comprehensive search, combining terms pertaining to sexual violence (eg, *sexual abuse*, *rape*, *sexual harassment*) with those of randomized clinical trials (eg, *RCT*, *randomized*). The full search strategy is included in the eMethods in the Supplement. Second, we searched additional web-based platforms, such as the ClinicalTrials.gov database and Google Scholar to check for potentially eligible papers. Third, the reference lists of all potentially included articles were further searched. Fourth, updated searches were conducted through December 2021.

Studies were selected independently by 2 trained student assistants. Titles and relevant abstracts were reviewed after removal of duplicates. The full texts of the remaining records were retrieved to determine inclusion in the meta-analysis. Two reviewers (A.P. and E.J.) performed quality cross-checks of the screenings and assessed eligible articles to ensure that they met criteria for inclusion. In cases of multiple publications from a single study, the article that provided the most comprehensive data was selected.

### Inclusion and Exclusion Criteria

Studies were selected if they (1) were randomized trials; (2) examined the efficacy of a psychosocial prevention program in reducing any type of sexual violence, such as sexual harassment, unwanted touching, or rape; (3) the program was compared with a control group, which could include no intervention, wait-list, or active controls (ie, minimal interventions, lectures on health, usual practice); and (4) was delivered to adolescents. Adolescents were defined as middle or high school students or individuals aged 10 to 19 years, as described by the World Health Organization.^15^ We excluded reviews, qualitative studies, nonrandomized studies, quasi-experimental studies, and articles that were not published in peer-reviewed journals. We also excluded studies or group comparisons that (1) were not psychosocial programs, such as those based only on cash transfers or on the legislation of sexual-related crimes; (2) did not follow up with the same cohort of participants; (3) compared different prevention programs with no control group; (4) targeted college or university students; (5) assessed sexual violence outcomes by behavioral counts or through official records; or (6) did not include enough information to compute effect sizes and raw data could not be retrieved after contacting the authors of the study.

### Data Extraction and Risk of Bias

Data concerning study information, outcomes, sample, and prevention program characteristics were independently extracted following a standardized form by 2 trained student assistants and 1 reviewer (A.P.). Data concerning effect sizes were extracted by 1 reviewer (A.P.) and cross-checked by an additional reviewer (H.M.F.). The risk of bias (ROB) of each included study was assessed separately by two reviewers (A.P. and H.M.F.) using the revised Cochrane collaboration’s tools for randomized trials.^16^

### Statistical Analysis

Pairwise meta-analyses were performed on experience (ie, being the target of) and perpetration of sexual violence. A meta-analysis was also conducted on a composite measure (ie, any perpetration or experience of sexual violence) involving all trials.

The odds ratio (OR) was calculated as an effect-size measure to reflect the difference between the prevention program group and control group at posttest or follow-ups. Since most of the included studies assessed sexual violence as a dichotomous outcome, the OR was chosen to limit the number of conversions to a common measure.

Very few trials reported follow-up after 2 years. Thus, we limited the analyses to a maximum length of follow-ups to 2 years. If a trial had several follow-up assessments, the mean OR was calculated using the method for multiple outcomes within a study described by Borenstein et al,^17^ which considers the correlation between different data gathered on the same participants. The same method was applied when combining different measures of sexual violence from the same study. Furthermore, if a trial had more than 2 arms, effect sizes of the different experimental conditions were combined with methods to avoid unit-of-analysis error.^18^

ORs based on the intention-to-treat (ITT) principle were preferred over non-ITT or per-protocol analyses and adjusted analyses were preferred over unadjusted analyses. However, we used unadjusted ORs for sensitivity analysis when available.

If a study did not report the OR, available data were used to calculate the effect size. Effect sizes for categorical data were calculated by using the exact number of participants with an event of sexual violence relative to the total number of participants randomized to that group. Cohen *d* was computed for continuous data and then converted to the OR.^17^

Effect sizes were pooled using random-effects models to incorporate the heterogeneity of the differences across the studies. Tests were 2-tailed and significance levels for statistical tests were set at *P* < .05. Between-study heterogeneity was measured using the *I^2^* statistic.^19^

To investigate sources of heterogeneity and identify characteristics of studies associated with effect sizes, we conducted exploratory subgroup and metaregression analyses. These analyses were done on the composite variable of any sexual violence. Subgroup analyses were conducted using a mixed-effects model on categorical moderators that included: income classification of the country according to the World Bank^20^ and coded as high- vs low- and middle-income countries; setting (school vs other), gender-specific program (eg, programs targeted at girls vs others), implementation-time (schooling hours vs nonschooling hours), delivery of programs (eg, adult-led vs other programs), comparison condition (active control vs waiting list or no program); fidelity to the program (moderate or high vs other); mean age (10-14 years vs 15-19 years); and risk of bias (high vs other). Additionally, 1 combination of categorical moderators was examined (school and age 15-19 years vs all others). Metaregression was applied to continuous covariates (eg, length, number of sessions, length of follow-up).

Publication bias was assessed through the funnel plot^21^ and testing for asymmetry using the Egger test statistic.^22^ We also used Duval and Tweedie trim and fill method^23^ to compute the adjusted effect size in case of publication bias. Further sensitivity analyses were performed by serially excluding each study to evaluate the impact of each individual study for the pooled effect size. All analyses were done with the Comprehensive Meta-Analysis software version 3.3.070 (Biostat). Data were analyzed from January to February 2022.

## Results

### Search Results and Study Characteristics

We screened 3335 titles and abstracts, removed 2515 (863 were duplicates and 1652 were not relevant), and subsequently assessed 820 full-text articles. Twenty-one studies met the inclusion criteria, and 20^24,25,26,27,28,29,30,31,32,33,34,35,36,37,38,39,40,41,42,43^ provided data for calculating effect sizes (eFigure 1 in the Supplement). We could not retrieve the necessary data to compute the effect size for 1 study,^44^ and this was subsequently excluded.

The 20 studies involved a total of 37 294 adolescents. Selected characteristics of the included programs are presented in Table 1. Eleven studies (55%) were conducted in North America,^24,28,29,32,33,34,38,39,40,42,43^ 2 (10%) in Europe,^27,35^ and 7 (35%) in sub-Saharan Africa.^25,26,30,31,36,37,41^ The mean age of participants ranged from 11 to 17.6 years old. Fifteen studies (75%) were school-based prevention programs and 5 (25%) were conducted in different settings, such as at a community agency.^33,36,37,39,41^ Four studies (20%) involved programs targeted at girls,^25,36,40,41^ 3 (15%) involved programs targeted at boys,^32,33,34^ and 13 (65%) involved mixed samples. The prevention programs ranged in length from 1 day to almost 2 years, and the number of sessions ranged from 1 to 32.

**Table 1.** Characteristics of Studies Included in the Meta-analysis

Source	Country	Mean age, y	No. of Participants^a^	Sample	Program characteristics
Connolly et al,^24^ 2015	Canada	12.4	509	Mixed	School-based youth-led psychoeducational program on bullying and dating aggression
Decker et al,^25^ 2018	Malawi	16.2	7832	Girls	School-based empowerment self-defense training
Devries et al,^26^ 2017	Uganda	13.2	3706	Mixed	School-based psychoeducational program on violence for staff and students to create a better learning environment
de Lijster et al,^27^ 2016	Netherlands	14.4	815	Mixed	School-based youth- and adult-led psychoeducational program on sexual harassment
Espelage et al,^28^ 2014	USA	11	3658	Mixed	School-based psychoeducational program on violence, including empathy training and communication skills
Foshee et al,^29^ 2005	USA	13.8	2344	Mixed	School-based psychoeducational program on dating violence norms and conflict management
Jemmott et al,^30^ 2018	South Africa	12.4	1057	Mixed	School-based psychoeducational program on sexual risk behaviors and gender/rape beliefs linked to forced sex
Mathews et al,^31^ 2016	South Africa	13	3451	Mixed	School-based psychoeducational program on sexual risk behaviors and violence
Miller et al,^32^ 2012	USA	15.5^b^	2006	Boys	School-based psychoeducational program, led by coaches who teach boys about healthy dating relationship
Miller et al,^33^ 2020	USA	15.5	866	Boys	Community-based psychoeducational program on masculinity norms, healthy relationships and sexuality
Miller et al,^34^ 2020	USA	13.4^b^	973	Boys	School-based psychoeducational program, led by coaches who teach boys about gender-based violence
Muck et al,^35^ 2021	Germany	14.2	681	Mixed	School-based psychoeducational program on sexual violence
Ozler et al,^36^ 2020	Liberia	13.5^b^	1216	Girls	Empowerment program including life-skills and violence training, caregiver sessions and savings start-up
Palermo et al,^37^ 2021	Tanzania	16	904	Mixed	Empowerment program including life-skills/sexual behaviors/violence training, mentoring and conditional grants
Peskin et al,^38^ 2019	USA	12.2	1760	Mixed	School-based psychoeducational program on dating violence
Rothman et al,^39^ 2020	USA	17.6	220	Mixed	Health care-based brief motivational interview on dating violence
Rowe et al,^40^ 2015	USA	15.6	83	Girls	Empowerment self-defense training
Stark et al,^41^ 2018	Ethiopia	14.6	919	Girls	Psychoeducational program on gender-based violence and life-skills training
Taylor et al,^42^ 2010	USA	12^b^	1639	Mixed	School-based psychoeducational program on dating violence and harassment
Taylor et al,^43^ 2013	USA	12^b^	2655	Mixed	School-based psychoeducational program on dating violence and harassment

^a^ Randomized or at baseline.

^b^ Approximated from available data.

Concerning the ROB assessment (eFigure 2 in the Supplement), 10 studies (50%) were estimated to have some concerns related to the randomization process (eg, not providing details on the procedure). Nine trials (45%) were judged as having some concerns due to deviations from the intended program (eg, changes in recruitment or implementation of the program), and 1 study (5%) was evaluated as high ROB. One study (5%) was evaluated as having some concerns in dealing with missing data (eg, lack of intention-to-treat-analysis), and 7 studies (35%) were evaluated as high ROB. Two trials (10%) were rated as having some concerns with the measurement of the outcome (eg, difficulty in blinding the intervention), and 2 (10%) were rated as high ROB. Finally, 13 (65%) trials presented some concerns in relation to the domain of selection of the reported results (eg, lack of protocol preregistration).

### Meta-analysis Main Outcomes

#### Perpetration of Sexual Violence

The forest plot for the 12 studies reporting outcomes for sexual violence perpetration is shown in the eFigure 3 in the Supplement. The pooled OR for these studies was 0.83 (95% CI, 0.73-0.95; *P* = .005), in favor of the prevention group as compared with the control group, with low or moderate heterogeneity (*I^2^* = 28.4%) (Table 2).

**Table 2.** Meta-analysis Main Outcomes

Variable	Trials, No. (%)	Participants No.	Odds ratio (95% CI)^a^	*I*^2^, %	*P* value
Perpetration of sexual violence	12 (60)	18 674	0.83 (0.73-0.95)	28.4	.005
Experience of sexual violence	16 (80)	33 229	0.87 (0.78-0.98)	45.1	.02
Any sexual violence	20 (100)	37 294	0.87 (0.78-0.97)	39.4	.009

^a^ According to the random-effects model.

#### Experience of Sexual Violence

Sixteen studies provided a measure for experience of sexual violence (eFigure 4 in the Supplement). The pooled OR yielded a significant association for the prevention group 0.87 (95% CI, 0.78-0.98; *P* = .02), with moderate heterogeneity (*I^2^* = 45.1%) (Table 2).

#### Any Sexual Violence

The Figure shows the forest plot for the effect sizes of all 20 studies reporting either a measure of perpetration, experience, or both. Sexual violence was significantly lower in the prevention group (OR, 0.87; 95% CI, 0.78-0.97; *P* = .009), compared to the control group. Heterogeneity was moderate (*I^2^* = 39.4%) (Table 2). According to the sensitivity analysis, in which the meta-analysis was serially repeated after exclusion of each trial, the results showed that no individual study significantly influenced the pooled effect size. An additional sensitivity analysis was performed using unadjusted ORs, and the results did not change.

**Figure.**
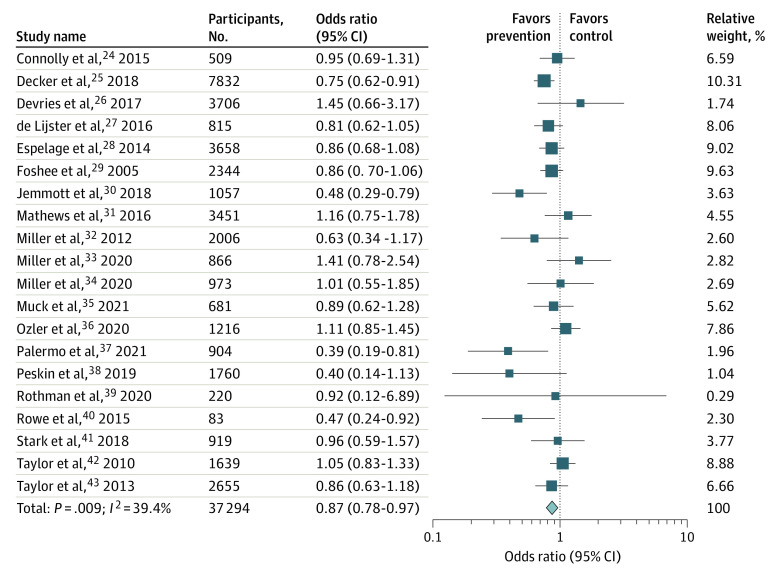
Efficacy of Prevention Programs on Any Sexual Violence

### Subgroup and Metaregression Analyses

Exploratory subgroup (Table 3) and metaregression (Table 4) analyses were based on the composite variable for any sexual violence. The analysis of subgroup contrasts, based on categorical moderators including income of the country, age, setting, gender-targeted program, implementation-time, delivery of programs, comparison condition, program fidelity, and risk of bias did not show statistically significant differences. Although not statistically significant, programs implemented with high fidelity, programs delivered in school or delivered to older adolescents had larger effect sizes. Furthermore, the combination of setting and age showed significantly larger associations for programs delivered both in school and with older adolescents aged 15 to 19 years vs other programs (Cochran *Q* = 4.8; *P* = .03). The pooled effect sizes were also regressed on continuous moderators (ie, length of program, number of sessions, length × sessions interaction, length of follow-up, and risk of bias), but no significant associations emerged.

**Table 3.** Subgroup Analyses

Variable	Trials, No. (%)^a^^,^^b^	OR (95% CI)^c^	*I*^2^, %	Cochran *Q*	*P* value^d^
Country					
High income	13 (65)	0.88 (0.80-0.97)	2.1	0.1	.73
Low or middle income	7 (35)	0.84 (0.64-1.10)	68		
Age, y					
10 to 14	13 (65)	0.91 (0.82-1.01)	26.5	1.8	.18
15 to 19	7 (35)	0.74 (0.56-0.98)	44		
Programs					
Gender-targeted	7 (35)	0.88 (0.69-1.11)	52.7	0	.90
Mixed samples	13 (65)	0.86 (0.76-0.98)	35.5		
Programs targeted at girls	4 (20)	0.84 (0.62-1.12)	65.2	0.1	.79
Programs targeted at boys or mixed samples	16 (80)	0.87 (0.77-0.99)	33		
Programs targeted at boys	3 (15)	0.97 (0.62-1.54)	41.9	0.3	.60
Programs targeted at girls or mixed samples	17 (85)	0.86 (0.77-0.96)	41.7		
Delivered by					
Adults	17 (85)	0.85 (0.75-0.97)	45	0.2	.68
Youth and adults	3 (15)	0.90 (0.73-1.11)	6.9		
Professionals	3 (15)	0.83 (0.59-1.15)	66.1	0.1	.79
Trained personnel	16 (80)	0.87 (0.76-0.99)	40.6		
Setting					
School	15 (75)	0.85 (0.76-0.94)	31.6	0.3	.57
Nonschool settings (e.g. community agencies)	5 (25)	0.95 (0.65-1.37)	51.5		
Delivered during class	9 (45)	0.86 (0.77-0.97)	24.6	0.0	.99
Delivered outside of schooling hours or in nonschool settings	10 (50)	0.86 (0.69-1.07)	52.7		
Fidelity					
Low or not reported	16 (80)	0.88 (0.78-0.99)	37.9	0.4	.54
Moderate or high	4 (20)	0.77 (0.53-1.13)	57.6		
ROB					
At least 1 domain with high ROB	10 (90)	0.88 (0.74-1.05)	36.7	0.1	.77
No domains with high ROBr	10 (90)	0.85 (0.74-0.99)	45.7		
Control condition					
Active control	7 (35)	0.83 (0.71-0.97)	37.8	0.4	.55
No program or waitlist	13 (65)	0.89 (0.76-1.03)	39.5		
Setting and Age					
School and ≥15 y	3 (15)	0.72 (0.60-0.85)	0	4.8	.03
Nonschool settings or <15 y	17 (85)	0.91 (0.81-1.01)	32.4		

Abbreviations: OR, odds ratio; ROB, risk of bias.

^a^ Analyses on “any sexual violence.”

^b^ Total number varies due to exclusion of trials in the subgroup comparison (eg, information not available).

^c^ According to the mixed-effects model.

^d^ Between-group difference.

**Table 4.** Metaregression

Variable	Coefficient (95% CI)^a^^,^^b^	*P* value
No. of domains with low ROB	0 (–0.13 to 0.14)	.95
No. of sessions^c^	0.01 (–0.02 to 0.04)	.52
Length of program	–0.01 (–0.09 to 0.07)	.87
Length × No.of sessions^c^	0 (–0.01 to 0)	.69
Length of follow-up	0 (–0.03 to 0.03)	.99

Abbreviation: ROB, risk of bias.

^a^ According to the random-effects model.

^b^ Analyses on “any sexual violence.”

^c^ One study was excluded from the analysis due to lack of data on program intensity.

### Publication Bias

Inspection of the funnel plot did not suggest publication bias for any of the outcomes analyzed (eFigure 5, 6, and 7 in the Supplement). This was supported by the trim-and-fill test, which did not identify any potential missing study, and the Egger test, which was not significant.

## Discussion

The aim of this study was to conduct a global evaluation of the efficacy of prevention programs for adolescent sexual violence. Our review included 20 RCTs that involved more than 37 000 adolescents. Results from the meta-analysis indicated that, overall, psychosocial prevention programs were associated with significant reductions of sexual violence during adolescence (eg, perpetration or experience of any sexual violence). Pooled effect sizes were in the small range, which is nonetheless meaningful when considered from a public health perspective with a focus on violence.^45^ Furthermore, these findings are promising, given the conclusions from previous literature reviews.^12,13^ Review articles about the prevention of sexual violence that focused primarily on college-aged youth and older individuals consistently described a lack of compelling evidence for program associations on self-reported experience and perpetration of sexual violence.^46,47,48^ Thus, sexual violence prevention programs targeted at adolescents may have a greater impact than programs targeted at older individuals, such as college students or adults.

We further investigated potential sources of heterogeneity in subgroup and metaregression analyses. Results showed that prevention programs delivered both in a school setting and targeting adolescents aged 15 to 19 years yielded significantly larger effect sizes, compared with programs delivered in other contexts (eg, community agencies) or involving younger adolescents. This finding is consistent with a previous meta-analysis on teen dating violence,^12^ which reported larger effect sizes for older adolescents. The trajectory of sexual violence, which tends to increase as adolescents become older, may help explain this finding.^6^ Programs offered prior to high school may require longer follow-up periods to detect associations due to the relatively low prevalence of sexually violent behaviors among middle schoolers. It is also possible that older adolescents may be more ready than younger ones to learn and apply information from sexual violence prevention programs because the content may be more relevant and meaningful to them. Additionally, programs delivered in a school setting may underscore for adolescents the importance of the material and help avoid stigmatization.^49^

Another important finding concerned the quality assessment of the included trials. Specifically, biases due to difficulty in blinding the intervention type, lack of preregistrations, absence of a detailed randomization procedure, and lack of intention-to-treat analysis were commonly observed. The delivery and evaluation of large-scale randomized prevention programs entail substantial challenges. Future research studies should include rigorous designs and methods to limit the influence of potential biases.

### Limitations

This study has several limitations, which should be considered when interpreting its findings. Although we implemented a broad literature search and checked full-text articles that reported on violence across adolescence, we may have missed trials that included sexual violence as a secondary outcome. Furthermore, we were not able to calculate the effect size for 1 study, which was excluded from the quantitative analysis. Another limitation pertains to the definition and assessments of sexual violence, which varied across studies, and prevented us from conducting analyses on specific sexually violent behaviors (eg, rape, sexual harassment). For this reason, we could only evaluate the efficacy of programs on general sexual violence outcomes, which were defined to include a broad range violent acts. Additionally, the programs differed greatly in approach and content, and program descriptions did not always provide sufficient information on theory of change or specific activities. As a result, comparing programs based on approach and content could not be done. Lastly, the subgroup and metaregression analyses were exploratory, and some subgroups included data from a small number of trials. As more data accumulate, future evaluations may more readily detect program characteristics that are important correlates of outcomes.

## Conclusions

The present research is the first meta-analysis that systematically assessed the efficacy of prevention programs for sexual violence during adolescence through a rigorous evaluation of RCTs. Despite the global burden and negative impacts of sexual violence in children and adolescents,^7,8^ the number of evidence-based prevention programs remains scant in comparison to other similarly important public health prevention targets such as bullying,^50^ obesity,^51^ mental health,^52^ and substance use.^53^ Evidence from this meta-analysis indicated that, overall, prevention programs were associated with reducing both the perpetration and experience of sexual violence during adolescence. It is also noteworthy that programs implemented in school settings and targeting older adolescents appeared to be more valuable. These findings have important implications for public health policies, with reference to the choice of setting and timing of program delivery. Nonetheless, findings also showed that the magnitude of effect sizes was small, and several studies presented some concerns of bias. Given the concerns of risk of bias across trials, further high-quality research is needed.

## Notes

Supplement.
eMethods.
**eFigure 1.** Flowchart of Included Studies **eFigure 2. **Risk of Bias Assessment **eFigure 3. **Efficacy of Prevention Programs on Perpetration of Sexual Violence **eFigure 4. **Efficacy of Prevention Programs on Experience of Sexual Violence **eFigure 5. **Funnel Plot for Perpetration of Sexual Violence **eFigure 6.** Funnel Plot for Experience of Sexual Violence **eFigure 7.** Funnel Plot for Any Sexual Violence
