# Supplementary material for: Assessment of Psychosocial Programs to Prevent Sexual Violence During Adolescence: A Systematic Review and Meta-analysis
Source: JAMA Netw Open. 2022 Nov 8;5(11):e2240895. doi: 10.1001/jamanetworkopen.2022.40895 (PMC9644260; doi:10.1001/jamanetworkopen.2022.40895)
Supplement: Supplement. — eMethods. eFigure 1. Flowchart of Included Studies eFigure 2. Risk of Bias Assessment eFigure 3. Efficacy of Prevention Programs on Perpetration of Sexual Violence eFigure 4. Efficacy of Prevention Programs on Experience of Sexual Violence eFigure 5. Funnel Plot for Perpetration of Sexual Violence eFigure 6. Funnel Plot for Experience of Sexual Violence eFigure 7. Funnel Plot for Any Sexual Violence [file jamanetwopen-e2240895-s001.pdf]

Supplemental Online Content

Piolanti A, Jouriles EN, Foran HM. Assessment of psychosocial programs to prevent sexual violence during adolescence: a systematic review and meta-analysis. *JAMA Netw Open*. 2022;5(11):e2240895. doi:10.1001/jamanetworkopen.2022.40895

eMethods.

eFigure 1. Flowchart of Included Studies

eFigure 2. Risk of Bias Assessment

eFigure 3. Efficacy of Prevention Programs on Perpetration of Sexual Violence

eFigure 4. Efficacy of Prevention Programs on Experience of Sexual Violence

eFigure 5. Funnel Plot for Perpetration of Sexual Violence

eFigure 6. Funnel Plot for Experience of Sexual Violence

eFigure 7. Funnel Plot for Any Sexual Violence

This supplemental material has been provided by the authors to give readers additional information about their work.

© 2022 Piolanti A et al. *JAMA Network Open.*

eMethods.

**Literature Search**	
PubMed	(“Sex Offenses” [Mesh] OR "Rape"[Mesh] OR “Sexual Violence” OR “Sexual Harassment” [Mesh] OR “Sexual Abuse”) AND ("Program Evaluation"[Mesh] OR Randomized or Randomized Trial or RCT)<br><br>Filter: Randomized Controlled Trials
PsycINFO/Eric/PsycArticles	(Sexual Abuse.sh OR Sexual Violence.mp OR Rape.sh OR Sexual Abuse.mp OR Sexual Harassment.sh) AND (Randomized.mp OR Randomized Trial.mp or RCT.mp)
Web Of Science	(TS=(“Sexual Violence” OR “Sexual Abuse” OR Rape OR “Sexual Harassment”) OR TI=(“Sexual Violence” OR “Sexual Abuse” OR rape OR “Sexual Harassment”) OR AB=(“Sexual Violence” OR “Sexual Abuse” OR Rape OR “Sexual Harassment”)) AND (TS=(Randomized or RCT OR “Randomized Trial”) OR TI=(Randomized OR RCT OR “Randomized Trial”) OR AB=(Randomized OR RCT OR “Randomized Trial”))

© 2022 Piolanti A et al. *JAMA Network Open.*

eFigure 1. Flowchart of Included Studies

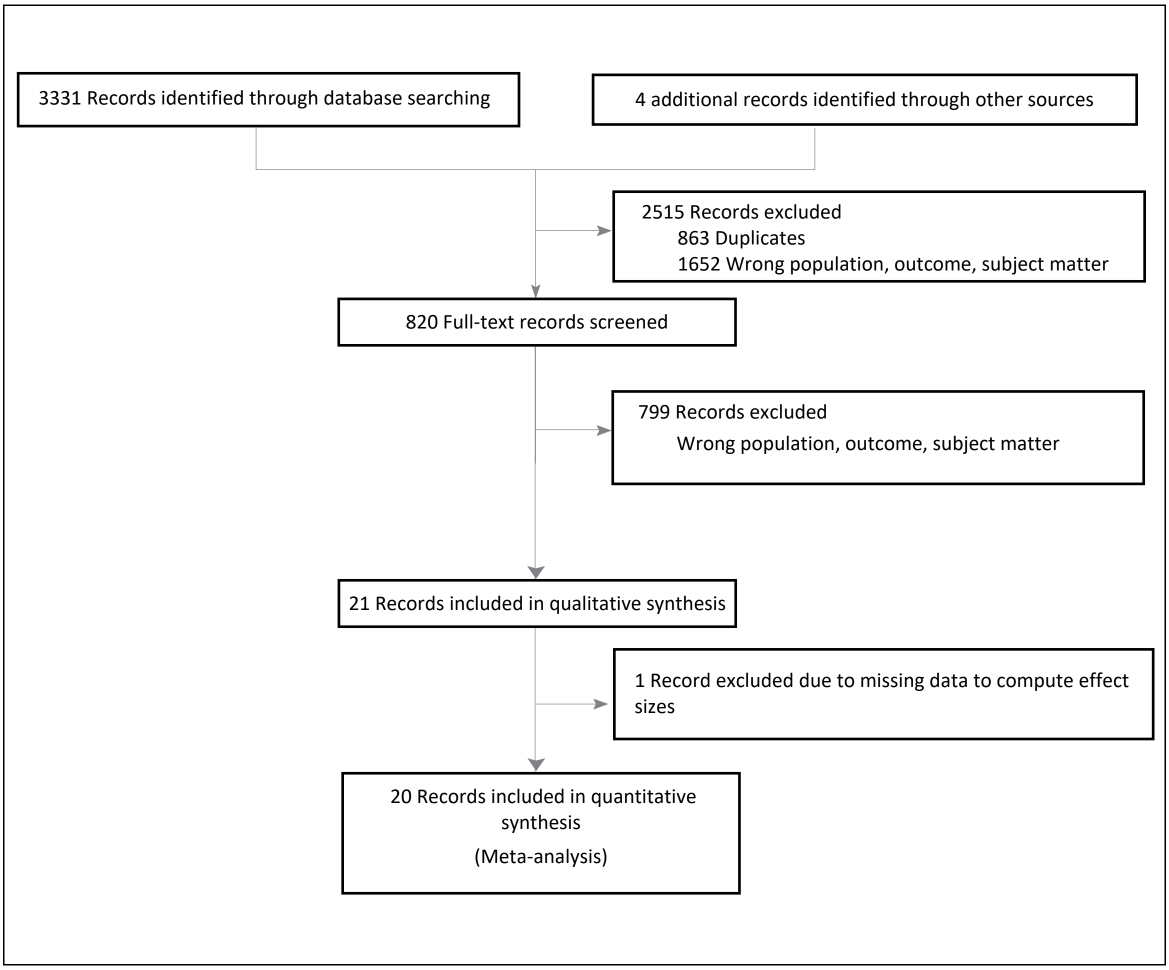

© 2022 Piolanti A et al. *JAMA Network Open.*

eFigure 2. Risk of Bias Assessment

Risk of bias domains

Study	D1	D2	D3	D4	D5
Connolly et al, 2015	-	+	+	+	-
Decker et al, 2018	-	+	X	X	+
Devries et al, 2017	+	-	+	-	+
de Lijster et al, 2016	-	-	+	-	-
Espelage et al, 2014	+	-	+	X	-
Foshee et al, 2005	+	+	-	-	-
Jemmott et al, 2018	+	+	+	+	-
Mathews et al, 2016	-	-	+	-	-
Miller et al, 2012	+	+	X	-	+
Miller et al, 2020a	-	X	+	+	+
Miller et al, 2020b	-	-	+	-	+
Muck et al, 2021	-	-	X	-	-
Ozler et al, 2020	-	-	+	-	+
Palermo et al, 2021	-	-	X	-	-
Peskin et al, 2019	-	+	X	-	-
Rothman et al, 2020	+	+	X	+	-
Rowe et al, 2015	+	+	+	-	-
Stark et al, 2018	-	-	+	+	+
Taylor et al, 2010	+	+	X	+	-
Taylor et al, 2013	-	+	X	+	-

Domains:
D1: Bias arising from the randomization process.
D2: Bias due to deviations from intended intervention.
D3: Bias due to missing outcome data.
D4: Bias in measurement of the outcome.
D5: Bias in selection of the reported result.

Judgement
X High
- Some concerns
+ Low

© 2022 Piolanti A et al. *JAMA Network Open.*

eFigure 3. Efficacy of Prevention Programs on Perpetration of Sexual Violence

Study name	N	Odds ratio (95% CI)	Relative Weight
de Lijster et al,[27] 2016	815	0.76 (0.58 – 0.99)	13.43
Espelage et al,[28] 2014	3658	0.81 (0.65 - 1.01)	16.20
Foshee et al,[29] 2005	2344	0.80 (0.64 - 0.99)	16.73
Jemmott et al,[30] 2018	1057	0.42 (0.24 - 0.73)	4.63
Miller et al,[32] 2012	2006	0.63 (0.34 - 1.17)	3.77
Miller et al,[33] 2020	866	1.41 (0.78 - 2.54)	4.11
Miller et al,[34] 2020	973	1.01 (0.55 - 1.85)	3.89
Muck et al,[35] 2021	681	0.94 (0.65 - 1.36)	8.62
Peskin et al,[38] 2019	1760	0.49 (0.18 - 1.34)	1.54
Rothman et al,[39] 2020	220	0.92 (0.12 - 6.89)	0.40
Taylor et al,[42] 2010	1639	1.03 (0.81 - 1.31)	15.14
Taylor et al,[43] 2013	2655	0.86 (0.64 - 1.16)	11.56
OR; *P* = .005; I^2 = 28.4%	18674	0.83 (0.73 - 0.95)	

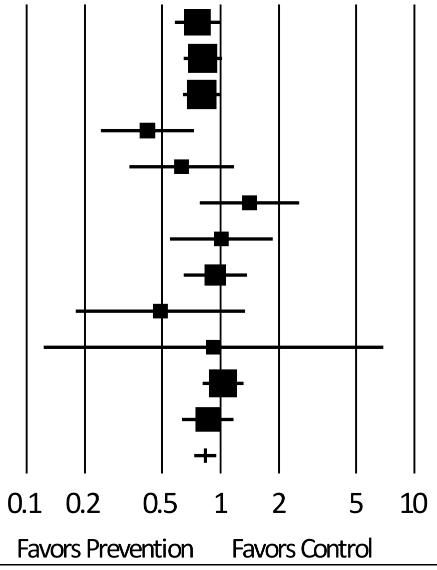

© 2022 Piolanti A et al. *JAMA Network Open.*

eFigure 4. Efficacy of Prevention Programs on Experience of Sexual Violence

Study name	N	Odds ratio (95% CI)	Relative Weight
Connolly et al,[24] 2015	509	0.95 (0.69 - 1.31)	7.18
Decker et al,[25] 2018	7832	0.75 (0.62 - 0.91)	11.08
Devries et al,[26] 2017	3706	1.45 (0.66 - 3.17)	1.93
de Lijster et al,[27] 2016	815	0.86 (0.66 - 1.12)	8.70
Espelage et al,[28] 2014	3658	0.91 (0.72 - 1.15)	9.65
Foshee et al,[29] 2005	2344	0.93 (0.75 - 1.15)	10.32
Jemmott et al,[30] 2018	1057	0.54 (0.33 – 0.87)	4.28
Mathews et al,[31] 2016	3451	1.16 (0.75 – 1.79)	4.96
Muck et al,[35] 2021	681	0.85 (0.59 – 1.22)	6.21
Ozler et al,[36] 2020	1216	1.11 (0.85 – 1.45)	8.53
Palermo et al,[37] 2021	904	0.39 (0.19 – 0.81)	2.17
Peskin et al,[38] 2019	1760	0.32 (0.11 – 0.94)	1.09
Rowe et al,[40] 2015	83	0.47 (0.24 – 0.92)	2.54
Stark et al,[41] 2018	919	0.96 (0.59 – 1.57)	4.15
Taylor et al,[42] 2010	1639	1.06 (0.84 – 1.34)	9.55
Taylor et al,[43] 2013	2655	0.86 (0.64 – 1.16)	7.66
OR; *P* = .02; I^2 = 45.1%	33229	0.87 (0.78 – 0.98)	

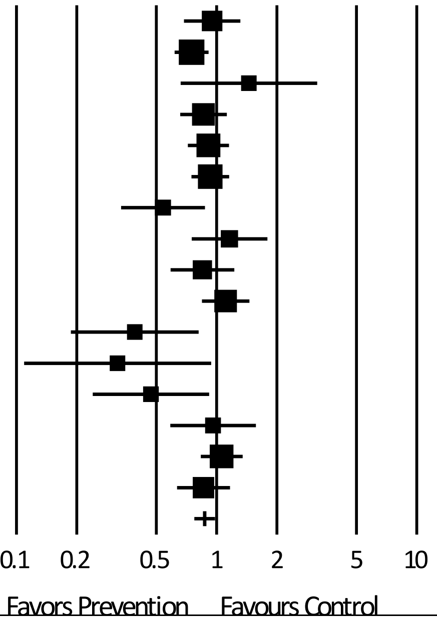

© 2022 Piolanti A et al. *JAMA Network Open.*

eFigure 5. Funnel Plot for Perpetration of Sexual Violence

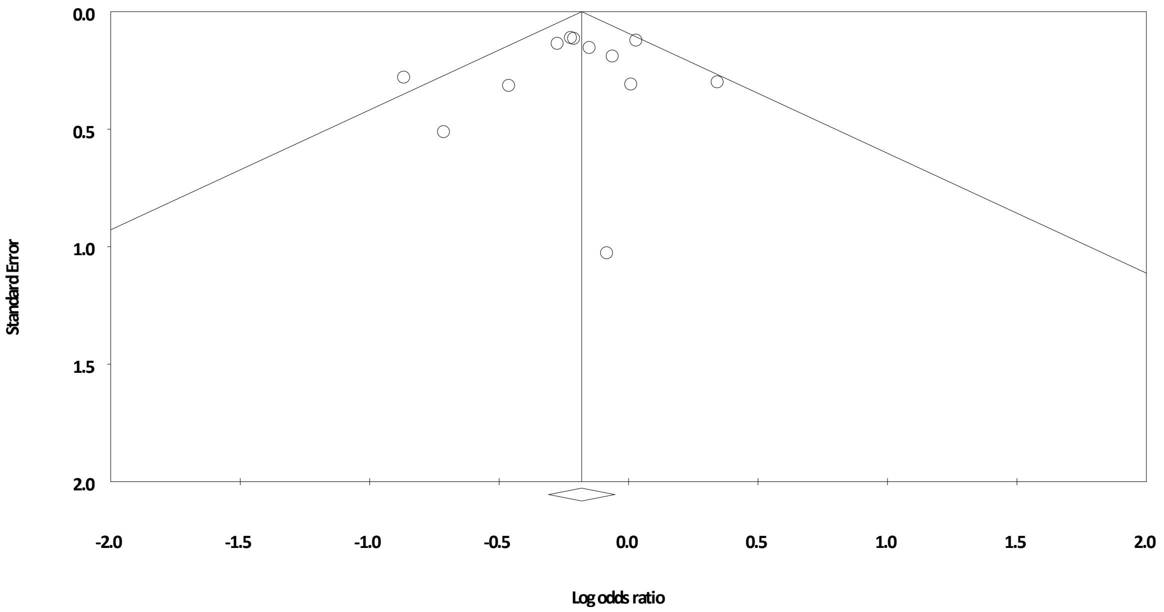

© 2022 Piolanti A et al. *JAMA Network Open.*

eFigure 6. Funnel Plot for Experience of Sexual Violence

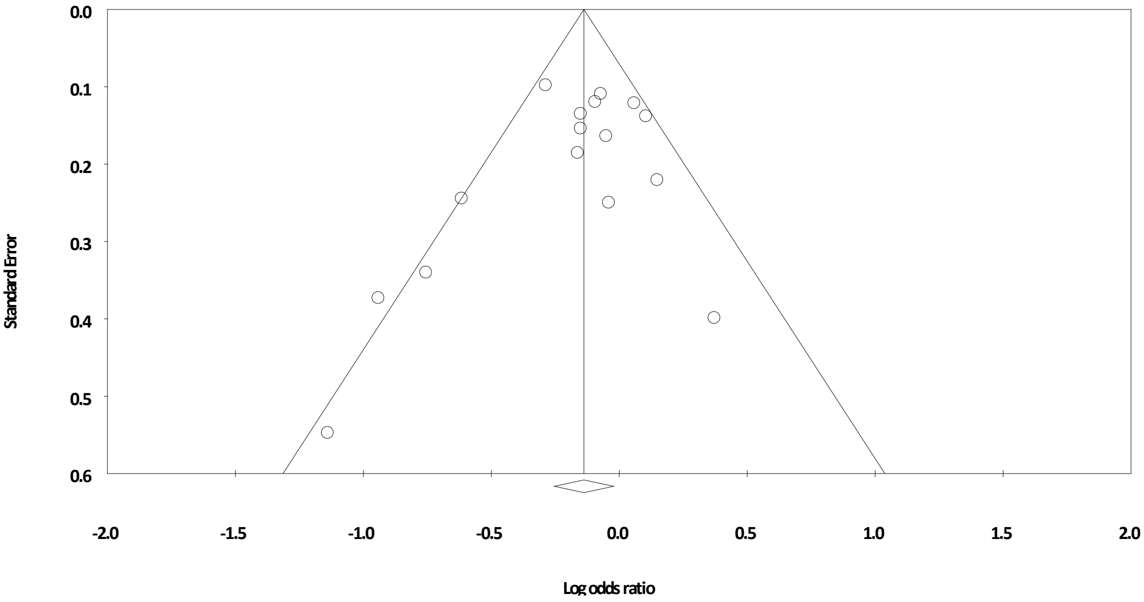

© 2022 Piolanti A et al. *JAMA Network Open.*

eFigure 7. Funnel Plot for Any Sexual Violence

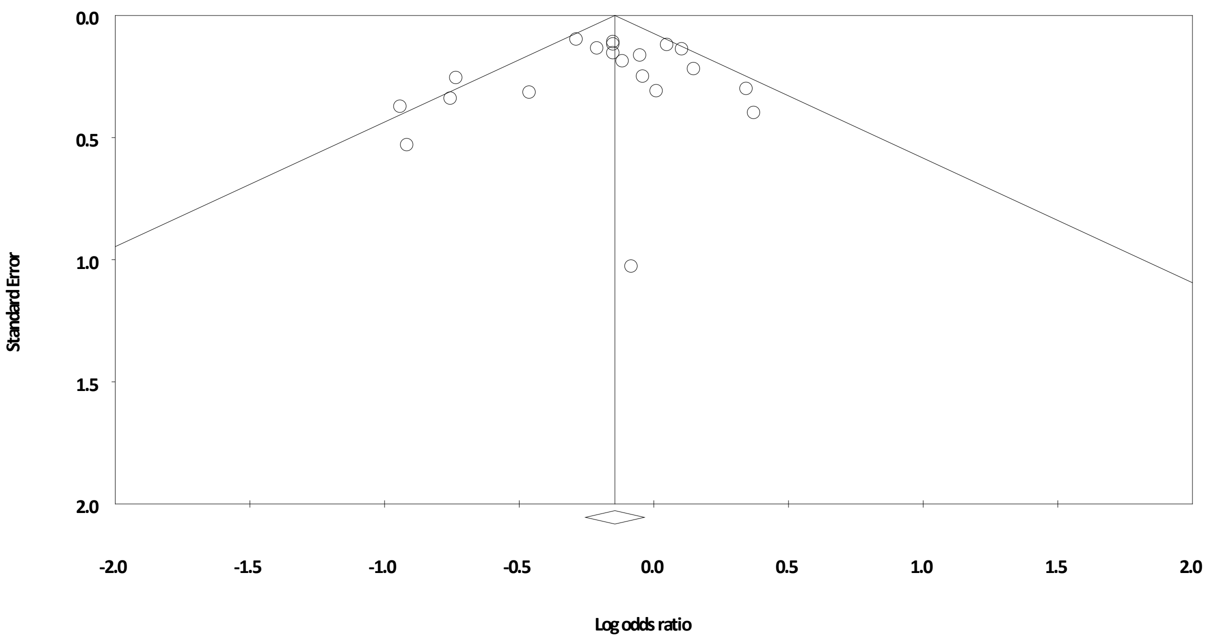

© 2022 Piolanti A et al. *JAMA Network Open.*
